# Total Ankle Replacement Through a Lateral Transfibular Approach in Patients with Ipsilateral Knee Arthrodesis: Report of Two Cases

**DOI:** 10.3390/jcm15062094

**Published:** 2026-03-10

**Authors:** Carla Carfì, Serban-Andrei Constantinescu, Cristian Indino, Federico Della Rocca, Camilla Maccario, Federico Giuseppe Usuelli

**Affiliations:** 1 Department of Biomedical Sciences, Humanitas University, 20090 Milan, Italy; 2 IRCCS Humanitas Research Hospital, Rozzano, 20089 Milan, Italy; 3 Ankle and Foot Unit, Humanitas San Pio X Hospital, 20159 Milan, Italy; serbanandreiconstantinescu@gmail.com (S.-A.C.); cristian.indino@gmail.com (C.I.); camillamaccario@gmail.com (C.M.); fusuelli@gmail.com (F.G.U.)

**Keywords:** arthrodesis, knee joint, arthroplasty, replacement, ankle, gait, biomechanical phenomena

## Abstract

**Background**: Knee arthrodesis markedly alters lower limb biomechanics and creates a challenging scenario when associated with end-stage ankle osteoarthritis. No prior reports have specifically described treatment with total ankle replacement (TAR) in the presence of an ipsilateral fused knee. This study evaluated the feasibility and mid-term outcomes of TAR in this rare condition. **Methods**: Two patients with post-traumatic end-stage ankle osteoarthritis and long-standing knee arthrodesis underwent TAR using a lateral transfibular approach with a Zimmer Trabecular Metal™ implant. Surgical planning aimed to restore coronal and sagittal alignment. Postoperative management and rehabilitation were specifically adapted to the absence of knee motion, with emphasis on gait re-education. Clinical and radiographic follow-up was performed up to 36 months. **Results**: At final follow-up, both patients showed substantial pain reduction, improved ankle range of motion, and recovery of a stable, functional gait compatible with knee fusion. Imaging demonstrated well-aligned, stable components without loosening or subsidence. No major complications or reoperations occurred. **Conclusions**: Lateral transfibular TAR appears feasible and effective for end-stage ankle osteoarthritis in patients with ipsilateral knee arthrodesis, preserving ankle motion and supporting functional ambulation in this complex setting.

## 1. Introduction

Knee arthrodesis is a limb-salvage procedure indicated for patients with a severely damaged, non-reconstructible knee joint, and represents a valid alternative to above-knee amputation (AKA) [1]. Common indications include failed total knee arthroplasty, periarticular tumors, post-traumatic arthritis, and chronic septic arthritis [2]. As a consequence of fusion, knee arthrodesis profoundly alters gait dynamics, typically resulting in a shortened stance phase, a prolonged swing phase, and increased step time on the operated side [3]. Compared with the contralateral non-operated limb, significant differences have been observed in hip, knee, and ankle motion, with compensatory adaptations that increase mechanical stress on adjacent joints during ambulation [2,3,4].

The coexistence of knee pathology and concomitant ipsilateral ankle disorder represents a complex and relatively underexplored topic, with only a few studies addressing their reciprocal biomechanical relationship [5,6,7], mainly focusing on how ankle pathology may influence the knee. However, no reports in the literature describe the reverse scenario—how knee fusion may accentuate or lead to ankle pathology—likely due to the rarity of this condition.

Historically, ankle arthrodesis has been considered the standard treatment for end-stage ankle osteoarthritis. Although effective in relieving pain, ankle fusion is associated with well-known limitations, including altered gait mechanics with reduced stride length and increased load transfer to adjacent joints, potentially leading to secondary degeneration over time [8]. Over the past two decades, advances in implant design and improved understanding of tibiotalar biomechanics have progressively shifted the standard of care toward total ankle replacement (TAR), with modern prostheses aiming to preserve joint motion, restore kinematics, and reduce compensatory overload on neighboring structures [9]. Nevertheless, overlap still exists between arthrodesis and TAR indications, as severe deformity, major bone loss, or complex multiplanar malalignment may make prosthetic implantation technically demanding, and in selected cases ankle arthrodesis may still be preferred [10].

Importantly, ankle arthrodesis represents a relative or absolute contraindication in specific suprasegmental conditions, including the presence of an ipsilateral total knee arthroplasty and, even more critically, knee fusion. In these scenarios, eliminating ankle motion may severely compromise global lower-limb biomechanics and functional ambulation. Preservation of ankle motion therefore becomes theoretically crucial in patients with a fused knee, where compensatory mechanisms at adjacent joints are already limited [11].

More recently, the introduction of the lateral transfibular approach for TAR has expanded the indications for prosthetic treatment, allowing the management of severe deformities previously considered unsuitable for arthroplasty. This approach provides direct visualization of the ankle center of rotation, facilitating accurate correction of coronal and sagittal plane deformities [12]. In patients with knee arthrodesis, whose gait is intrinsically altered, restoration of ankle kinematics may support improved biomechanical adaptation and a gait pattern closer to physiological function.

To our knowledge, no previous case reports have described the management of ankle osteoarthritis in the setting of ipsilateral knee arthrodesis. Such cases represent a challenging scenario that requires meticulous preoperative planning, tailored surgical techniques, and individualized rehabilitation protocols to achieve optimal lower limb alignment and minimize postoperative complications or the need for reoperation [5].

We present two cases of end-stage post-traumatic ankle osteoarthritis in patients with long-standing ipsilateral knee arthrodesis, both treated with total ankle replacement (TAR) through a lateral transfibular approach.

## 2. Case Reports

### 2.1. Case One

A 55-year-old woman was referred to our department for evaluation of severe right ankle pain. She was previously known to our service for post-traumatic ankle osteoarthritis, having been assessed three years earlier. At that time, given an acceptable functional reserve, she was managed conservatively with a combination of physical therapy, custom insoles, and anti-inflammatory medications. She has since reported progressive worsening of pain over the past six months, which had increasingly limited her walking ability, significantly impaired her daily activities, and necessitated daily pain medication intake (Visual Analog Scale, VAS 10/10). Her past medical history included a giant cell tumor of the proximal right tibia diagnosed 35 years earlier, which was treated with surgical resection and ipsilateral knee arthrodesis using an Ilizarov frame. The disease remained in complete remission following treatment. In 1989, she sustained a right ankle fracture on the same limb, which was treated conservatively. She had no known comorbidities or history of chronic medical conditions.

At presentation, her weight was 56 kg, height 165 cm, and Body Mass Index (BMI) 20.6 kg/m^2^.

On physical examination, the patient ambulated with the right lower limb held in extension due to the knee arthrodesis and demonstrated an antalgic gait. The ankle was aligned in varus, with reduced stability during single-leg stance. Ankle range of motion was markedly limited, with 15° of fixed equinus and plantarflexion of 20°, and a dorsiflexion deficit resulting from both joint stiffness and partial tibialis anterior insufficiency. The foot demonstrated a cavus morphology. Passive range of motion testing through inversion and eversion of the patient’s foot showed that her subtalar motion was reduced compared to the contralateral side but remained preserved and asymptomatic.

**Figure 1:**
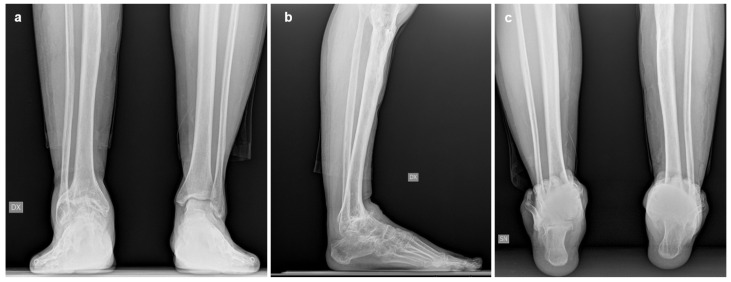
Preoperative weight-bearing radiographs of Case one: (**a**) anteroposterior view; (**b**) lateral view; (**c**) Saltzman view.

**Figure 2:**
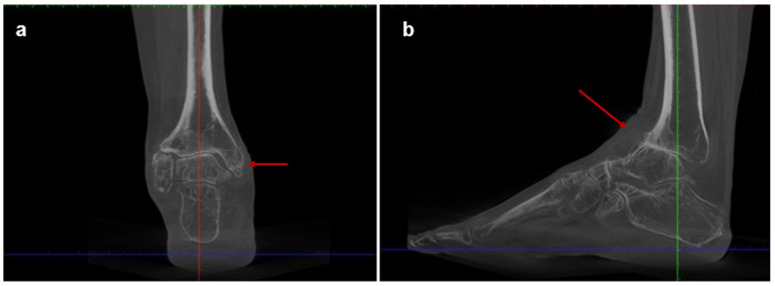
Preoperative weight-bearing computed tomography (WBCT) scans of Case one: (**a**) coronal and (**b**) sagittal WBCT views demonstrating advanced tibiotalar osteoarthritis and distal tibial bone irregularities. The arrow in panel (**a**) indicates advanced tibiotalar osteoarthritis, while the arrow in panel (**b**) indicates anterior talar shift.

Imaging assessment included conventional bilateral foot and ankle weight-bearing radiographs in anteroposterior, lateral, and Saltzman views (Figure 1), as well as bilateral Weight-Bearing CT (WBCT) scans (Figure 2). Imaging demonstrated advanced right ankle osteoarthritis (Kellgren–Lawrence grade 4 [13]) with severe distal tibial bone deformity.

Hindfoot and ankle alignment were fixed in varus and equinus, with an anterior shift in the talus relative to the tibia. Radiographic measurements are summarized in Table 1.

The patient underwent TAR through a lateral transfibular approach, performed according to the originally described technique [14], using a Zimmer Trabecular Metal™ Total Ankle Replacement (Zimmer TM TAR, Zimmer Biomet, Warsaw, IN, USA), implant (size 3 with a size 2 polyethylene insert). The fibula was shortened and stabilized with five 3.5 mm cortical screws. Prophylactic fixation of the medial malleolus was achieved with a single 4.5 mm In2Bones^®^ screw. Additionally, a percutaneous first tarsometatarsal (TMT1) arthrodesis in elevation, according to the Lapidus technique, was performed to correct plantarflexion using an 8 × 40 mm In2Bones^®^ screw. The procedure was carried out under regional anesthesia with sedation, following preoperative antibiotic prophylaxis with cefazolin 2 g.

Postoperatively, the patient was placed in a short-leg cast and instructed to maintain partial weight-bearing for two weeks, progressing to full weight-bearing for an additional two weeks. She was discharged on postoperative day two in good general condition. The cast was removed at four weeks, after which a rehabilitation program was initiated, including triceps surae stretching, calf muscle strengthening, and proprioceptive training. Targeted assisted physiokinesitherapy was strongly recommended to emphasize gait re-education, given the challenges posed by the presence of ipsilateral knee arthrodesis.

**Figure 3:**
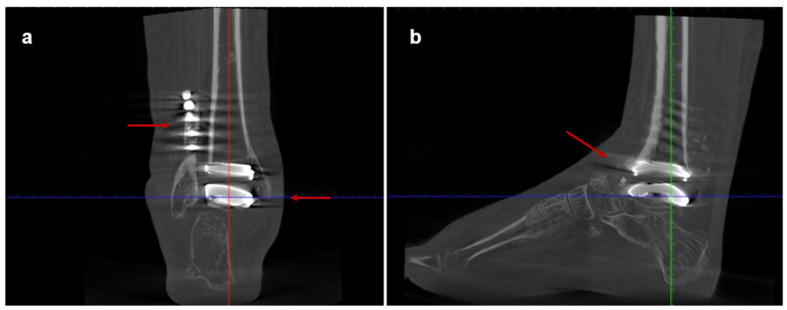
Postoperative weight-bearing computed tomography (WBCT) scans of Case one: (**a**) coronal and (**b**) sagittal views at 3-year follow-up. The arrow in panel (**a**) indicates correct implant positioning and stable fixation, while the arrow in panel (**b**) indicates absence of radiographic signs of loosening.

At the 6-month follow-up, the patient had resumed her normal daily activities. Clinically, she demonstrated a smooth gait compatible with the ipsilateral knee fusion, with the hindfoot and ankle well aligned and stable. Ankle range of motion had improved to 40° of plantarflexion and 10° of dorsiflexion. Imaging confirmed a well-fixed and osseointegrated prosthesis with excellent ankle alignment (Figure 3). Post-operative radiographic measurements are summarized in Table 1. At the 36-month follow-up, imaging showed a stable implant with normal alignment. Clinically, the patient remained asymptomatic and pain-free (VAS 1/10), with maintained function and gait stability. She was independently ambulatory without walking aids and fully independent in activities of daily living. The patient gave informed consent for the publication of her clinical details and images.

### 2.2. Case Two

A 69-year-old man presented to our department with a 15-year history of progressive left ankle pain. At the time of evaluation, pain was present during weight-bearing, walking, and even at rest (VAS 8/10). His past medical history included a left ankle fracture in 1996, treated conservatively with casting. In 1972, he sustained an intra-articular fracture of the distal left femur, which was surgically treated but subsequently complicated by chronic osteomyelitis. The therapeutic course was completed in 1976 with a left fibular autograft and ipsilateral knee arthrodesis. He had a history of arterial hypertension, which was currently well controlled with pharmacological therapy.

On examination, he was 168 cm tall, weighed 72 kg, with a BMI of 25.5 kg/m^2^. The ankle was rigid, with limited range of motion (5° dorsiflexion and 10° plantarflexion), and pain localized to the tibiotalar joint during weight-bearing and ambulation. Consistent with his previous fusion, he ambulated with a plantigrade foot but with the knee in extension, and the ankle showed a valgus alignment. Subtalar range of motion was reduced.

**Figure 4:**
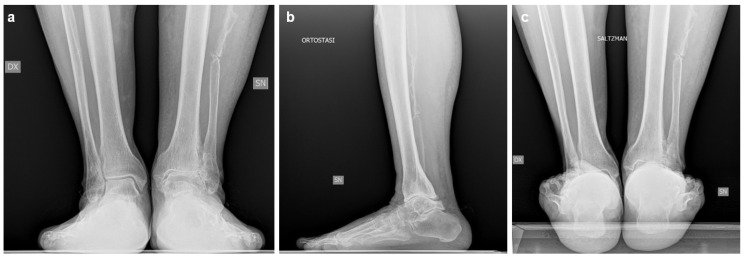
Preoperative weight-bearing radiographs of Case two: (**a**) anteroposterior view; (**b**) lateral view; (**c**) Saltzman view.

**Figure 5:**
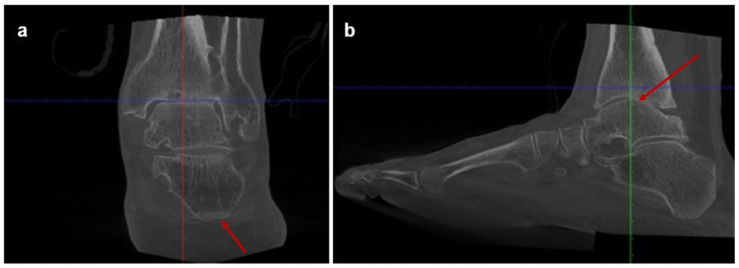
Preoperative weight-bearing computed tomography (WBCT) scans of Case two: (**a**) coronal and (**b**) sagittal views showing severe tibiotalar osteoarthritis. The arrow in panel (**a**) indicates valgus malalignment, while the arrow in panel (**b**) indicates posterior talar displacement.

Imaging, including bilateral weight-bearing X-rays (Figure 4) and WBCT (Figure 5), demonstrated end-stage tibiotalar osteoarthritis (Kellgren–Lawrence grade 4 [13]) with distal tibial and distal fibular bone deformity. The ankle showed a valgus alignment with posterior shift in the talus. Early degenerative changes were also observed in the talonavicular and subtalar joints. The midshaft of the fibula displayed residual deformity from previous surgical procedures. Radiographic measurements are summarized in Table 2. The patient gave informed consent for the publication of his clinical details and images.

The patient underwent TAR through a lateral transfibular approach, carried out according to the originally described technique [14], with implantation of a Zimmer Trabecular Metal™ Total Ankle Replacement (Zimmer TM TAR), size 4 with a size 0 polyethylene insert. The fibula was restored to its appropriate length and stabilized with a single 3.5 mm cortical screw followed by fixation with a six-hole Zimmer ULS plate. The procedure was performed under regional anesthesia with sedation, following administration of preoperative antibiotic prophylaxis with 2 g of cefazolin.

Postoperatively, given the patient’s good bone quality and overall clinical condition, a quick recovery protocol, compared to most postoperative aftercare regimens, was applied [15]. A short-leg cast was placed, and immediate full weight-bearing with crutches was allowed. He was discharged on postoperative day two in good general condition. At three weeks, the cast was removed and sutures were taken out, after which a rehabilitation program was initiated, including triceps surae stretching, calf muscle strengthening, proprioceptive training, and hydrotherapy. Considering the concomitant knee arthrodesis, particular attention was devoted to the recovery of a functional and efficient gait pattern, supported by targeted assisted physiokinesitherapy.

**Figure 6:**
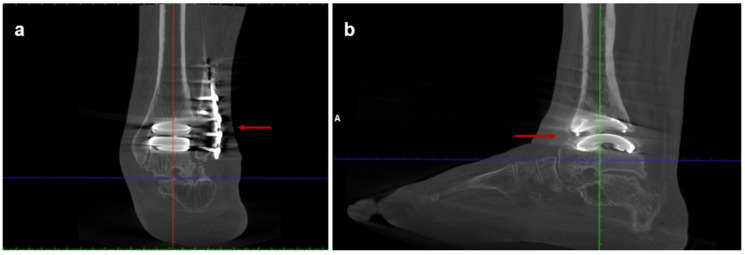
Postoperative coronal (**a**) and sagittal (**b**) WBCT views at 3-year follow-up of Case two. The arrow in panel (**a**) indicates restoration of alignment, while the arrow in panel (**b**) indicates absence of radiographic signs of loosening.

The patient was reviewed at 6, 12, 24 and 36 months postoperatively. Clinically, he demonstrated progressive improvement in gait pattern—consistent with his previous knee fusion—and ankle range of motion, achieving a painless 10° dorsiflexion and 30° plantarflexion (VAS 0/10). Imaging at each follow-up showed a stable implant with correct alignment and no evidence of subsidence (Figure 6). Post-operative radiographic measurements are summarized in Table 2. By the last follow-up, the patient was independently ambulatory without assistive devices and fully independent in activities of daily living. The patient gave informed consent for the publication of his clinical details and images.

## 3. Discussion

The most important finding of this report is that, despite the biomechanical challenges posed by ipsilateral knee arthrodesis, total ankle replacement (TAR) through a lateral approach achieved excellent mid-term clinical and radiographic outcomes. In both patients, TAR allowed restoration of ankle alignment, preservation of residual motion, and recovery of a stable and functional gait compatible with knee fusion. The traditional alternative to total ankle replacement for the treatment of end-stage ankle osteoarthritis is ankle arthrodesis. Comparative studies have shown no consistent differences between TAR and ankle arthrodesis with respect to patient-reported outcome measures (PROMs), as both procedures significantly improve symptoms compared with the preoperative condition [16]. In other words, both interventions are effective in reducing pain and improving function. However, reported short-term complication rates at approximately two years are higher after ankle arthrodesis (about 27%) than after TAR (about 16%), while infection rates appear similar (around 4%). Published revision rates are also higher after ankle arthrodesis compared with TAR (approximately 16% versus 11%) [16]. Ankle arthrodesis was considered suboptimal in these patients, as further rigidifying the lower limb and its restriction of rotational movements would have exacerbated biomechanical limitations, impacting hip kinematics. In patients with a functional knee, part of this rotational demand can be compensated at the knee joint; however, this compensatory mechanism is lost in the presence of knee arthrodesis. From a purely biomechanical standpoint, an alternative staged strategy could consist of conversion of knee arthrodesis to total knee arthroplasty followed by surgical management of the ankle with arthrodesis or total ankle replacement [17]. However, conversion of long-standing knee fusion is a technically demanding procedure and may be associated with substantial soft-tissue balancing difficulties, uncertain musculotendinous functional recovery, and increased infectious risk in post-septic cases, with variable functional outcomes after prolonged compensatory gait adaptation [18]. Therefore, this approach may not be appropriate or advantageous in all patients and should be considered on an individual basis. Consequently, TAR represents the only viable option for preserving a near-physiological gait pattern in these patients, reinforcing its role as a joint-preserving procedure.

The management of lower limb disorders involving combined deformities at multiple levels (hip, knee, and ankle) remains complex, with heterogeneous and often inconclusive evidence in the literature. Knee arthrodesis, in particular, substantially alters gait mechanics and overall lower limb function; indeed, bilateral involvement or pre-existing ipsilateral hip fusion is generally considered a contraindication for knee fusion [2]. According to the same principle, ankle arthrodesis was deemed an unfavorable option in our patients. Some authors have attempted to clarify the biomechanical interplay between these joints through experimental and clinical studies. Naylor et al. [5] investigated the relative influence of the knee and ankle in coronal plane deformity in knee osteoarthritis, highlighting how the hindfoot can compensate for malalignment if healthy, but fails to do so when stiff, particularly during the stance and propulsive phases of gait. Stevens et al. [19] examined pathological lower limb biomechanics in patients with hip pathology, either weak or stiff, and reported a redistribution of power among lower extremity joints with altered ankle mechanics, emphasizing the need to preserve ankle function in this population. Roney et al. [7] analyzed knee kinetics and kinematics in patients with prior ankle replacement or fusion; while no significant clinical differences were found, the study was biased by barefoot gait testing and a small sample size, highlighting the need for larger, long-term investigations.

Reports of concomitant deformity involving multiple lower limb joints are rare. Lesiak [20] described two cases of multi-apical tibial deformity involving both the knee and ankle, treated with a staged approach: initial ankle arthrodesis and tibial osteotomy for realignment, followed by total knee arthroplasty, achieving good clinical and radiological outcomes at 3-year follow-up. Pagenstert and Hintermann [21] reported a case of simultaneous bilateral total knee and TAR, highlighting the advantage of performing all joint replacements in a single session, allowing rehabilitation to be conducted in one phase without the limitation of other arthritic joints. Nozaka [22] presented a case of simultaneous total knee arthroplasty and ankle fusion in a patient with Charcot neuroarthropathy; this strategy proved effective in maintaining leg length and achieving satisfactory alignment.

To our knowledge, this is the first reported mini-series of TAR performed in patients with prior knee arthrodesis, and specifically, the first series describing TAR using a lateral surgical approach. In this scenario, the accuracy of rotational alignment during prosthesis implantation becomes paramount. The decision to employ a lateral transfibular approach and the selected implant type was guided by the intrinsic advantages of this technique. This approach provides direct visualization of the ankle’s center of rotation during the procedure, facilitating accurate correction of coronal plane deformities and, importantly, sagittal plane abnormalities. In particular, it enables restoration of the physiological center of rotation in patients with anterior talar translation, a frequent finding in individuals with knee arthrodesis and concomitant ankle osteoarthritis [12]. The fibular osteotomy performed in this approach further facilitates achieving the desired correction. In addition, the lateral approach requires less bone resection, reducing the risk of limb shortening and preserving bone stock for potential revision surgery. Finally, it minimizes the risk of wound complications or neurovascular injury, as it is performed at a distance from the main neurovascular bundle compared with the traditional anterior approach [14]. In both cases, clinical and radiographic outcomes were excellent at mid-term follow-up, consistent with the results reported in the literature about this type of implant [23,24].

Rehabilitation and gait re-education were more complex in this specific setting and had to be specifically adapted due to altered suprasegmental mechanics. At present, no specific or standardized rehabilitation protocols are available in the literature for patients undergoing total ankle replacement in the presence of ipsilateral knee arthrodesis. With regard to the early recovery pathway, both patients were included in a fast-track rehabilitation protocol based on risk stratification scores calculated according to published criteria [15]. The postoperative program therefore focused on early functional loading and task-oriented gait restoration rather than isolated recovery of joint mobility. Rehabilitation primarily targeted the recovery of a stable and efficient gait pattern under load, given the central role of ankle function in step progression and push-off mechanics. Targeted assisted physiokinesitherapy was centered on supervised gait training and functional weight-bearing activities. In the presence of ipsilateral knee arthrodesis, gait re-education was specifically directed toward safe limb advancement and step pattern regularization during early loading.

This report has several limitations. First, the number of cases is limited, preventing any generalization of the results. Second, no functional scores or formal gait analysis were performed, which would have provided objective measures of functional recovery. Third, although full-length weight-bearing radiographs were used in the clinical assessment of these patients, they were not available for inclusion in the manuscript, limiting comprehensive evaluation of global lower-limb mechanical alignment. Finally, although follow-up reached 3 years, this period is still relatively short to draw definitive conclusions regarding long-term implant survival and functional outcomes.

Nevertheless, the main strength of this report lies in being, to our knowledge, the first description of total ankle replacement performed in patients with ipsilateral knee arthrodesis and the first to report the use of this specific prosthetic implant in such a setting. These cases contribute original information regarding the feasibility and technical considerations of TAR in a rare and biomechanically demanding condition. From a clinical perspective, preserving ankle motion appears particularly relevant when knee mobility is absent, as the compensatory capacity of the lower limb is inherently reduced. In this context, TAR may be considered a joint-preserving strategy, provided that careful preoperative planning, accurate restoration of coronal, sagittal, and rotational alignment, and a rehabilitation program focused on gait re-education are implemented.

## 4. Conclusions

Total ankle replacements through a lateral transfibular approach can be a viable option for the management of end-stage ankle osteoarthritis in patients with ipsilateral knee arthrodesis, offering the benefit of motion preservation and satisfactory clinical and radiological outcomes when meticulous surgical planning and tailored rehabilitation are applied. Although staged conversion of knee arthrodesis to total knee arthroplasty followed by ankle surgery may represent a biomechanically appealing alternative, it is not universally applicable; in carefully selected patients with long-standing knee fusion, total ankle replacement may represent a reliable joint-preserving solution.

## Figures and Tables

**Table 1:** Preoperative and postoperative radiographic measurements of Case one.

	Pre-Operative	Post-Operative
Lateral Distal Tibial Angle (LDTA)	95.69°	90.74°
Tibio-Talar Surface angle (TTS)	79.38°	91.15°
Talar Tilt (TT)	2.56°	0.9°
Anterior Distal Tibial Angle (ADTA)	67.31°	79°
Sagittal Meary’s angle	+19°	+12°
Hindfoot Moment Arm	−5.6 mm	+8 mm

**Table 2:** Preoperative and postoperative radiographic measurements of Case two.

	Pre-Operative	Post-Operative
Lateral Distal Tibial Angle (LDTA)	87.62°	90°
Tibio-Talar Surface angle (TTS)	92.29°	91.13°
Talar Tilt (TT)	3.55°	0.4°
Anterior Distal Tibial Angle (ADTA)	75.67°	90.07°
Sagittal Meary’s angle	−13.29°	−9°
Hindfoot Moment Arm	+6 mm	+11 mm

## Data Availability

The raw data supporting the conclusions of this article will be made available by the authors on request.

## Abbreviations

The following abbreviations are used in this manuscript:

TAR	Total Ankle Replacement
BMI	Body Mass Index
WBCT	Weight-Bearing CT
